# Developmental Trajectories of Science Identity Beliefs: Within-Group Differences among Black, Latinx, Asian, and White Students

**DOI:** 10.1007/s10964-021-01493-1

**Published:** 2021-09-13

**Authors:** Kayla Puente, Christine R. Starr, Jacquelynne S. Eccles, Sandra D. Simpkins

**Affiliations:** 1 School of Education, University of California, Irvine, CA USA; 2 Institute for Positive Psychology and Education, Australian Catholic University, North Sydney, NSQ 2060 Australia

**Keywords:** Science, Identity, Adolescence, Race or Ethnicity, College generation

## Abstract

Though adolescents’ science identity beliefs predict positive STEM outcomes, researchers have yet to examine developmental differences within racial/ethnic groups despite theoretical arguments for such studies. The current study examined science identity trajectories for Black (14%), Latinx (22%), Asian (4%), and White (52%) students (*N* = 21,170; 50% girls) from 9^th^ grade to three years post-high school and the variability within each racial/ethnic group based on gender and college generational status. Contrary to the literature, students’ science identities increased over time, and the increases were larger for potential first- versus continuing-generation White students. Potential continuing-generation boys had stronger 9^th^ grade science identities than potential first-generation girls in all groups except Asians. The findings suggest who might benefit from additional supports within each racial/ethnic group.

## Introduction

Despite the increased attention to foster equity in science, there continue to be disparities (National Science Foundation, 2019). The existing literature has primarily focused on between-group racial/ethnic differences demonstrating that Black and Latinx students have lower academic performance, motivational beliefs, and persistence in science than White and Asian students (Estrada et al., 2017). Although these findings identify groups that could benefit from more support and have resulted in beneficial interventions (Hecht et al., 2019), focusing only on between-racial/ethnic group comparisons can also perpetuate deficit narratives by reinforcing negative stereotypes that people hold towards certain racial/ethnic groups (Beasley & Fischer, 2012). Studies on between-group differences also homogenize racial/ethnic groups despite evidence that there are often larger within- than between-group differences (Causadias et al., 2018). Complementary research needs to focus on the variability within each racial/ethnic group in order to identify those who are succeeding in science. This emphasis on within-group variability is crucial in preventing the erasure of marginalized groups (Cole, 2020; Syed et al., 2018) and helps counter common racial/ethnic stereotypes, including that Black and Latinx students do not want to pursue science. College generation status and gender could help identify within-group differences as these characteristics are associated with students’ pursuit of science and motivational beliefs, including science identity (Engle, 2007; Harackiewicz et al., 2016). According to the situated expectancy-value theory, students who identify with science are more likely to pursue science; thus, it is important to understand how intersecting background characteristics predict students’ science identity beliefs (Eccles & Wigfield, 2020). The current study addresses these gaps in the research by describing the developmental trajectories of science identity among Black, Latinx, Asian, and White students and the extent to which these trajectories vary by students’ college generation status and gender within each racial/ethnic group.

### Developmental Trajectories of Students’ Science Identity Beliefs

Adolescence is a time of identity development and exploration, including developing educational and occupational identities (Eccles & Wigfield 2020; Osborne & Jones, 2011). Although identity development begins in adolescence, scholars argue identity development and exploration of one’s occupational choices continue during emerging adulthood, which spans ages 18 to 25 years (Arnett, 2014). During these years, individuals transition from high school to college or work and explore a variety of occupational possibilities. Though scholars may debate on whether adolescence ends at 18 or in the mid 20’s, they agree that high school and the years following are central for individuals’ occupational identities. According to situated expectancy-value theory, individuals’ identity in a particular domain, like science, is an important determinant of what they pursue and their performance (Eccles & Wigfield, 2020; Osborne & Jones, 2011). Students’ science identity is positively correlated with their intent to pursue a career in STEM and actual persistence in STEM (Carlone & Johnson, 2007; Hazari et al., 2013). Thus, understanding how adolescents’ science identity changes over time is important to explore, particularly among groups of students traditionally underrepresented in STEM. Existing research on the development of individuals’ science identity beliefs (reviewed next) largely focuses on a select group, namely college students. Few studies chart science identity development during high school and for individuals who do not attend college.

Though the existing literature notes that youth’s motivational beliefs across a variety of domains decline during middle and high school (Wigfield & Cambria, 2010; Wigfield et al., 2006), a few recent studies suggest the developmental changes in science identity among college students depends on their gender and race/ethnicity (e.g., Aschbacher et al., 2010; Packard & Nguyen, 2003). Male undergraduate students, for example, were more likely to have high science identity beliefs that increased throughout their time in college whereas women began college with moderate science identity beliefs that remained stable (Robinson et al., 2018). Black and Latinx college students were more likely to begin college with low to moderate science identity beliefs which decreased over time compared to Asian and White students (Robinson et al., 2018). Additionally, situated expectancy-value theory also highlights how individuals’ science identity may be shaped by experiences they have, identifying either more or less with the domain over time (Eccles & Wigfield, 2020). More research is needed to understand development during high school and college and the transition across these two periods.

### Within Racial/Ethnic Group Variability: Gender and College Generation Status

Situated expectancy-value theory argues that individuals’ demographic characteristics, such as race/ethnicity and gender, shape their achievement-related outcomes through differential societal expectations, resources, and socialization processes (Eccles & Wigfield, 2020). In the U.S., for instance, the cultural construct of who a “scientist” is often includes men and Asian or White individuals (Miller et al., 2018; Starr, 2018). Individuals who fit this stereotype in terms of gender or race/ethnicity are more likely to see themselves and be seen as a scientist whereas women and underrepresented minorities encounter various barriers and challenges within the sciences, such as lack of access to advanced courses and discrimination (Grossman & Porche, 2014; Strayhorn et al., 2013). In addition to race/ethnicity and gender, situated expectancy-value theory argues that parent education shapes students’ outcomes through resources and socialization processes (e.g., gendered socialization; Eccles 2005). An indicator of parent education that gives a more contextualized experience is college generation status. First-generation college students are the first in their family to go to college and often face barriers and challenges, such as fewer family educational resources (Engle, 2007; Gibbons & Borders, 2010). There are substantial gaps between first- and continuing-generation college students pursuing science college majors (Harackiewicz et al., 2014). Thus, the intersection of gender and potential college generation status could help describe the rich diversity within racial/ethnic groups. The terms potential first- and continuing-generation college student are used in this paper to refer to high school students as they have the potential to be first-generation or continuing-generation college students.

Taken together, the separate literatures on gender and college generation status suggest that potential first-generation girls may have the lowest science identity beliefs whereas potential continuing-generation boys may have the highest science identity beliefs, with potential continuing-generation girls and potential first-generation boys in the middle. When looking at patterns by gender, girls and women on average have lower identity beliefs in science than boys and men (e.g., Huang, 2013; Snodgrass Rangel et al., 2020). This is related to societal factors, such as the prevalent stereotype that men rather than women are scientists (Miller et al., 2018), as well as other barriers such as gender bias and sexual harassment in the sciences (Leaper et al., 2012; Leaper and Starr, 2019). Meanwhile, first-generation college students face multiple barriers and challenges in college compared to their continuing-generation peers, such as poor academic preparation (Atherton, 2014; Harackiewicz et al., 2014) and underrepresentation in STEM college majors (Chen, 2005). At the high school level, potential first-generation college students are less likely to take advanced math and science courses (Gibbons & Borders, 2010) and have lower math and science motivational beliefs, including science identity, than potential continuing-generation peers (Snodgrass Rangel et al., 2020). However, one study found that the differences in students’ motivational beliefs became nonsignificant once race/ethnicity, gender, SES, and high school math achievement were controlled for (Snodgrass Rangel et al., 2020), further suggesting that these are significant intersections related to science identity beliefs that warrant investigation.

Although certain challenges may be common among some demographic groups, such as fewer educational resources and cultural mismatches for potential first-generation college students (Wilson & Kittleson, 2013), the differences across the four groups defined by gender and potential college generation status may depend on the racial/ethnic group. For example, although STEM gender gaps generally replicate across racial/ethnic groups, the gaps are sometimes larger among White and Latinx students than among Asian and Black students (Hsieh et al., 2021; for a review, see Parker et al., 2019). These differential patterns align with the literatures on Latinx, Asian, and Black students. For example, there are strong traditional gender roles within Latinx communities, which could be related to low representation of Latina women in STEM even though they have higher rates of entering college compared to Latino men (Flores, 2011). In contrast, research has found that Asian boys and girls may be more similar than different due to the model minority stereotype and the high value placed on STEM by Asian families (Min & Jang, 2015). The Black community in the U.S. often fosters a sense of self-reliance and strength among girls and women, which could lessen gender differences among Black students (Hanson, 2012). Moreover, the prevalence of college generation status varies systematically across racial/ethnic groups with 41% of Black and 61% of Latinx undergraduate students as first-generation compared to only 25% of White and Asian students (Postsecondary National Policy Institute, 2020). Thus, Black and Latina potential first-generation women are also likely to experience more barriers in science that can lead to weaker science identities due to the added underrepresentation of their race and/or college generation status (e.g., Harackiewicz et al., 2016; Kang et al., 2018). Though potential continuing-generation males should have stronger science identities compared to the other three groups, it is unclear if this pattern will emerge among Black and Latinx students given the negative stereotypes around Black and Latino boys and men (Musto, 2019). In sum, this body of research demonstrates that the rich diversity within each racial/ethnic group may be unique to each group and warrants investigation given the dearth of existing studies.

## Current Study

The literatures on science identity development over time as well as within racial/ethnic group differences remain scarce. To address these two gaps, the first research aim of this study is to describe the developmental trajectories of science identity beliefs for Black, Latinx, Asian, and White students separately from 9^th^ grade to three years post-high school. It is hypothesized that all students would have either stable or declining science identity beliefs over time. The second research aim investigated the extent to which the intersection of gender and potential college generational status within each racial/ethnic group predicts science identity developmental trajectories because of the need in the literature to consider the heterogeneity within racial/ethnic groups. It is expected that potential first-generation female students will have the lowest science identity beliefs in 9^th^ grade and experience weaker science identity beliefs over time within each racial/ethnic group compared to potential continuing-generation males, except for Asian students.

## Methods

### Participants

Participants were from the High School Longitudinal Study of 2009 (HSLS). HSLS is a nationally representative longitudinal study on adolescent STEM motivation and outcomes (for more information, see https://nces.ed.gov/surveys/hsls09/index.asp). The sample includes 944 high schools across the United States. On average, 27 students were selected from each school to participate in the study (Ingels et al., 2011). The HSLS dataset was designed to be a stratified, two-stage random sample design with schools as the primary sampling unit for the first stage and students randomly selected within schools via a stratified systemic sampling procedure during the second stage (for more information, see Duprey et al., 2018).

The analytic sample had 21,170 participants who were surveyed when students were in 9^th^ grade, in 11^th^ grade, and three years after their high school graduation. The analytic sample was 50% female, 46% potential first-generation college, 52% White non-Hispanic, 22% Latinx, 14% Black, and 4% Asian (Table 1). Potential first-generation college students accounted for 30–66% within each racial/ethnic group: 37% White students, 52% Black students, 66% Latinx students, and 30% Asian students (see Table 2 for percentage breakdown of intersection of gender and college generation status within each racial/ethnic group).

**Table 1:** Descriptive and correlational statistics of study variables (analytic sample)

Variable	1.	2.	3.	4.	5.	6.
1. Science identity (9^th^ grade)	1					
2. Science identity (11^th^ grade)	0.45***	1				
3. Science identity (3 years post-HS)	0.34***	0.50***	1			
4. Potential first-generation college	−0.13***	−0.11***	−0.07***	1		
5. Female students	−0.06***	−0.04***	−0.08***	0.00	1	
6. Science grade (8^th^ grade)	0.35***	0.24***	0.18***	−0.22***	0.07***	1
Full Analytic Sample						
Mean/ % (SE)	2.31 (0.01)	2.48 (0.01)	2.61 (0.01)	46% (0.01)	50% (0.01)	4.10 (0.02)
Range	1–4	1–4	1–4	0–1	0–1	1–5
% Missing	11%	5%	30%	0%	0%	14%
Specific Racial/Ethnic Groups	*M*(SE)	*M*(SE)	*M*(SE)	%	%	*M*(SE)
Black (14%)	2.16 (0.05)	2.34 (0.04)	2.57 (0.05)	52%	57%	3.87 (0.05)
Latinx (22%)	2.20 (0.03)	2.37 (0.03)	2.55 (0.03)	66%	49%	3.83 (0.04)
Asian (4%)	2.61 (0.06)	2.75 (0.06)	2.78 (0.06)	30%	47%	4.52 (0.05)
White (52%)	2.36 (0.01)	2.54 (0.01)	2.65 (0.01)	37%	48%	4.26 (0.01)

*Note*. Frequencies in the table are all weighted except for correlations

SOURCE: U.S. Department of Education, Institute of Education Sciences, National Center for Education Statistics, High School Longitudinal Study of 2009 (HSLS:09), Base Year, First Year Follow-Up, Second Year Follow-Up

*p < 0.05. **p < 0.01. *p < 0.001

**Table 2:** Descriptive statistics by racial/ethnic group

	Black	Latinx	Asian	White
*N*	2190	3380	1710	11,850
Female PFG students	30%	31%	10%	18%
Female PCG students	27%	18%	37%	30%
Male PFG students	23%	35%	19%	19%
Male PCG students	21%	16%	33%	33%

*Note*. Frequencies in the table are weighted. PFG = potential first-generation college students; PCG = potential continuing-generation college students

SOURCE: U.S. Department of Education, Institute of Education Sciences, National Center for Education Statistics, High School Longitudinal Study of 2009 (HSLS:09), Base Year, First Year Follow-Up, Second Year Follow-Up

Participants were excluded if they did not have information on college generational status, gender, or race/ethnicity as these were grouping variables in the analysis. When comparing the excluded (*n* = 4,040) and analytic (*n* = 21,170) samples, four out of five estimated comparisons were statistically significant, but only one difference had a small effect size (i.e., 8^th^ grade science grade, Cohen’*s d* = 0.42, see Appendix A). All other effect sizes were less than a small effect size (i.e., Cramer’s *V* ≤ 0.10 or Cohen’s *d* ≤ 0.20).

### Procedures and Measures

The data were collected during the fall of 9^th^ grade in 2009, the spring of 11^th^ grade (2012), and three years after the expected graduation year (2016). Data were primarily collected in schools during 9^th^ and 11^th^ grade (for more information, see Duprey et al., 2018). When students were out of high school, data were collected through either self-administered surveys using the internet, computer-administered telephone interviewing, or computer-assisted field interviewing (for more information, see Duprey et al., 2018).

#### Science identity

Science identity was measured using a 2-item average score reported by students for each time point when students were in 9^th^, 11^th^, and three years post-high school (Shanahan, 2009). Aligned with the definition of attainment value from situated expectancy-value theory and the conceptualization of identity work, science identity referred to the extent to which individuals’ saw themselves and felt they were seen by others as a science person (Calabrese Barton et al., 2013; Eccles & Wigfield, 2020). The science identity measure included the following two items: “You see yourself as a science person” and “Others see you as a science person.” These items were measured on a 4-point Likert scale that were reverse coded so that higher scores reflected greater identification as a “science person” (1 = *Strongly disagree*, 2 = *Disagree*, 3 = *Agree*, 4 = *Strongly agree*). These items had strong reliability at 9^th^ grade (Spearman-Brown *r*_*s*_ = 0.72, *p* < 0.001), 11^th^ grade (Spearman-Brown *r*_*s*_ = 0.79, *p* < 0.001), and three years post-high school (Spearman-Brown *r*_*s*_ = 0.83, *p* < 0.001) (Eisinga et al., 2013).

#### Race/ethnicity

To minimize missing data, the race/ethnicity composite variable was based on adolescent and parent reports created by NCES. Students noted their race/ethnicity by selecting a racial/ethnic category (e.g., American Indian/Alaska Native, Asian, Black/African American) during the base year and follow-up year.

#### Gender

Gender was measured dichotomously (1 = *Female students*, 0 = *Male students*). The variable used to measure gender was also a composite variable created by NCES. To eliminate any missingness, student report, parent report, and/or the school-provided sampling roster was used to measure gender.

#### Potential college generation status

Potential first-generation college students included students who did not have at least one parent with an associate degree or higher (Engle, 2007). This variable was coded dichotomously; those who did not have at least one parent with an associate’s degree or higher were coded as 1 (i.e., potential first-generation college students) whereas those with at least one parent with an associate’s degree or higher were coded as 0 (i.e., potential continuing-generation college student).

#### 8^th^ grade science course grade

Students’ final grade in their most advanced 8^th^ grade science course grade was utilized as a control for the current study (i.e., “What was your final grade in this science course?”). This item was adolescent-reported and was measured on a 5-point Likert scale that was reversed to have higher scores reflect higher science grades (1 = *Below D*, 5 = *A*).

### Data Analysis Plan

Data were analyzed in Mplus 8.0 (Muthén & Muthén, 2012). Because the dataset is based on a stratified, two-stage random sample design with strata, primary sampling units (PSUs), and sampling weights, the appropriate weights were utilized in the analyses to ensure findings are representative of the national population and to account for nonresponse. Thus, for each model, the TYPE = COMPLEX command was used to indicate the strata, primary sampling unit (i.e., schools), and the appropriate weight. The SUBPOPULATION command was utilized to separately analyze each of the four racial/ethnic groups.

Three separate series of measurement invariance tests were estimated of the 2-factor identity model over (a) time, (b) gender, and (c) potential college generational status within each racial/ethnic group to test if science identity had similar measurement properties across time and across the groups of interest (i.e., gender and college generational status) (see Appendix B; Grimm et al., 2017). Because the identity items were measured on 4-point scales, they were analyzed as categorical indicators in the measurement invariance models. For each analysis, configural, weak, and strong invariance model were tested (Grimm et al., 2017). Though experts agree that the change in chi-square or the DIFFTEST often has a high Type I error rate (Liu et al., 2017; Sass et al., 2014), particularly with large sample sizes as is the case here, there is less agreement on what other criteria to use in determining measurement invariance with categorical indicators. Some suggestions include using a change in CFI ≤ 0.01 (Jin, 2020) instead of the DIFFTEST or using additional criteria when the DIFFTEST is statistically significant, such as a change in RMSEA and change in CFI (Svetina and Rutkowski, 2017). Given the lack of consensus, a range of criteria were used to determine invariance. A nonsignificant DIFFTEST denoted invariance. If the DIFFTEST was statistically significant, two sets of criteria were used to determine invariance: (a) a change in CFI ≤ 0.01 (Jin 2020), and (b) a change in RMSEA ≤ 0.05 for weak invariance, and a change in RMSEA ≤ 0.01 coupled with a change in CFI ≤ 0.002 for strong invariance (Svetina and Rutkowski, 2017).

#### Hypothesis testing

For the first hypothesis, it was predicted that students’ science identity trajectories would remain stable or decline over time. Separate latent growth curves were estimated for each racial/ethnic group (Muthén and Muthén, 1998–2017). To identify the optimal growth model, a no-growth model (which only included the intercept) and a linear growth model (which included the intercept and linear slope; Grimm et al., 2017) were estimated. The optimal model was determined by assessing the fit of each model, the growth factors, and the change in chi-square between the two nested models. Model fit was assessed by evaluating the chi-square, standardized root mean square residual (SRMR), root mean square error of approximation (RMSEA), Tucker-Lewis index (TLI), and the comparative fit index (CFI) (Grimm et al., 2017). Models were considered acceptable if the following standards were met: a small chi-square, an SRMR less than 0.10, an RMSEA less than 0.08, and a CFI/TLI greater than 0.90 (Hu & Bentler, 1999).

For the second hypothesis, it was hypothesized that the intersection of gender and college generational status would predict differences in students’ science identity trajectories, such that potential first-generation female students would have the weakest science identity beliefs in 9^th^ grade and largest declines over time for each racial/ethnic group except for Asian students due to the minimal gender differences in the literature regarding this group (Hsieh et al., 2021). Dichotomous variables were created that represented possible intersections (i.e., female potential first-generation student, female potential continuing-generation student, male potential first-generation student, and male potential continuing-generation student). These four groups were compared in order to uncover the patterns associated with intersecting characteristics and science identity. To test this aim, the intercept and slope of students’ science identities were regressed onto three dichotomous codes representing the four groups defined by gender and college generation status. All pairwise comparisons across the four groups were calculated by re-estimating the models with a different reference group (e.g., male potential continuing-generation students were the reference group in one set of analyses). Students’ 8^th^ grade science course grade was used as a covariate in these analyses.

#### Missing data and robustness checks

Missing data were present in the current study due to the nature of longitudinal data (Young and Johnson 2015). Participants were compared within the analytic sample based on whether they had complete data (*n* = 13,190) or some missing data (*n* = 7,970). All eight comparisons were statistically significant but only one had at least a small effect size (see Appendix A). Specifically, participants with complete data were more likely to have higher science grades at 8^th^ grade compared to those with missing data (*t*[18,220] = 14.79, *p* < 0.001, Cohen’s *d* = 0.24). Full information maximum likelihood (FIML) was used to include participants with missing data (Enders, 2010).

Because scholars have different perspectives about the effect of estimating missing data for those who dropped out of a longitudinal study (Enders, 2010; Young & Johnson, 2015), a robustness check was conducted where all the main analyses were repeated with a sample that excluded participants who did not participate in the last time point (i.e., when students were three years post-high school). The total sample size for the robustness check was 14,890.

## Results

### Descriptive Statistics

Descriptive statistics are displayed in Table 1. In 9^th^ grade, students, on average, disagreed that they were or that others saw them as a science person. This average increased slightly for all groups in 11^th^ grade and three years post-high school, with some racial/ethnic groups almost reaching an average score of agreeing (i.e., a score of 3) that they viewed themselves as a science person (e.g., Asian students). Among the full analytic sample, science identities for each wave were positively associated with one another. For example, having a stronger science identity in 9^th^ grade relative to one’s peers was associated with a stronger science identity in 11^th^ grade (*r* = 0.45, *p* < 0.001). Female and potential first-generation students had lower science identity beliefs at each time point compared to their peers (College generation: *R* = −0.07–−0.13, *p* < 0.001; Gender: *R* = −0.04 - −0.08*, p* < 0.001).

### Developmental Trajectories of Black, Latinx, Asian, and White Students’ Science Identities

**Fig. 1:**
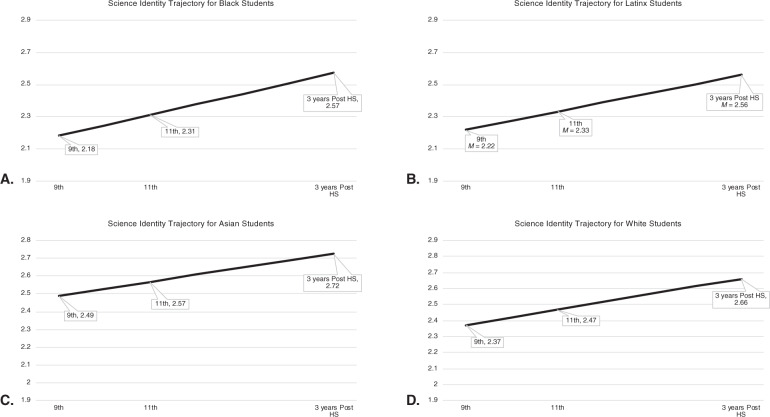
Linear Trajectories of Science Identity Beliefs by Race/Ethnic Group from 9^th^ Grade to 3 Years Post-High School. SOURCE: U.S. Department of Education, Institute of Education Sciences, National Center for Education Statistics, High School Longitudinal Study of 2009 (HSLS:09), Base Year, First Year Follow-Up, Second Year Follow-Up

Measurement invariance tests indicated that science identity beliefs evidenced full weak and full or partial strong invariance across time, gender, and college generational status within each racial/ethnic group (see Appendix B). To investigate the first research hypothesis, developmental trajectories were estimated separately for each racial/ethnic group. It was hypothesized that students’ science identity trajectories would remain stable or decline over time for each racial/ethnic group. Findings indicated that the linear growth model was a better fit than the no growth model in each racial/ethnic group (Δχ^2^ [Δdf] = 30.70 – 564.79 [3], *p* < 0.001; see Table 3 and Fig. 1).

**Table 3:** Model fit comparisons for the latent growth curve analyses of science identity by racial/ethnic group from 9^th^ grade to 3 years post-HS

*Black students’ science identity*											
Model	χ^2^	df	*p*	Δ χ^2^	Δdf	*p*	RMSEA	90% CI	CFI	TLI	SRMR
No growth	138.47	6	<0.001	—	—	—	0.10	0.09; 0.12	0.38	0.69	0.14
Linear*	21.34	3	<0.001	117.14	3	**<0.001**	0.05	0.03; 0.08	0.91	0.91	0.04
*Latinx students’ science identity*											
No growth	171.17	6	<0.001	—	—	—	0.09	0.08; 0.10	0.55	0.78	0.13
Linear*	25.96	3	<0.001	145.21	3	**<0.001**	0.05	0.03; 0.07	0.94	0.94	0.05
*Asian students’ science identity*											
No growth	45.75	6	<0.001	—	—	—	0.06	0.05; 0.08	0.75	0.88	0.10
Linear*	15.05	3	0.002	30.70	3	**<0.001**	0.05	0.03; 0.07	0.93	0.93	0.09
*White students’ science identity*											
No growth	718.35	6	<0.001	—	—	—	0.10	0.10; 0.11	0.65	0.83	0.10
Linear*	153.56	3	<0.001	564.79	3	**<0.001**	0.07	0.06; 0.07	0.93	0.93	0.05

*Note*. *Indicates the model with the better fit indices

SOURCE: U.S. Department of Education, Institute of Education Sciences, National Center for Education Statistics, High School Longitudinal Study of 2009 (HSLS:09), Base Year, First Year Follow-Up, Second Year Follow-Up

As shown in Fig. 1A, Black students on average had moderately weak science identities in 9^th^ grade (*B* = 2.18, SE = 0.03, *p* < 0.001) and demonstrated increases in their science identities over time (*B* = 0.07, SE = 0.01, *p* < 0.001). The model also suggested that there was significant variance around the intercept (*Variance* = 0.25, SE_variance_ = 0.03, *p* < 0.001), but not around the slope (*Variance* = 0.00, SE_variance_ = 0.00, *p* = 0.58). This suggests that Black students varied in terms of their science identity beliefs in 9^th^ grade, but there were minimal within-group differences in terms of the change over time.

The model for Latinx students suggested students on average also had moderately weak science identities in 9^th^ grade (*B* = 2.22, SE = 0.03, *p* < 0.001), but developed stronger science identities over time (*B* = 0.06, SE = 0.01, *p* < 0.001; Fig. 1B). There also was significant variance or within-group differences in terms of 9^th^ grade identities (*Variance* = 0.27, SE_variance_ = 0.03, *p* < 0.001), but not in terms of changes in their identities over time (*Variance* = 0.00, SE_variance_ = 0.00, *p* = 0.18).

The findings indicate that Asian students, on average, had slightly weak science identities in 9^th^ grade (*B* = 2.49, SE = 0.05, *p* < 0.001) and evidenced positive, significant increases over time (*B* = 0.04, SE = 0.01, *p* < 0.001; Fig. 1C). Lastly, there was significant variance or within-group differences for the intercept (*Variance* = 0.33, SE_variance_ = 0.05, *p* < 0.001), but not for the slope (*Variance* = 0.00, SE_variance_ = 0.00, *p* = 0.05).

White students on average had slightly weak science identity beliefs in 9^th^ grade (*B* = 2.37, SE = 0.01, *p* < 0.001) and demonstrated significant, positive changes over time (*B* = 0.05, SE = 0.00, *p* < 0.001; Fig. 1D). There was significant variance around the intercept (*Variance* = 0.33, SE_variance_ = 0.01, *p* < 0.001) and slope (*Variance* = 0.00, SE_variance_ = 0.00, *p* < 0.001). Because there were significant within-group differences for the intercept and slope, the relation between the intercept and slope was examined. White students’ 9^th^ grade science identity beliefs were negatively associated with the rate of change from 9^th^ grade to three years post-high school (*B* = −0.01, SE = 0.00, *p* < 0.001).

### The Intersection of Gender and College Generational Status within Each Racial/Ethnic Group

The second hypothesis was to test the extent to which the intersection of gender and college generational status predicted students’ science identity beliefs in 9^th^ grade and science identity development within each racial/ethnic group while controlling for students’ 8^th^ grade science course grade. It was hypothesized that potential first-generation female students would have the weakest science identity beliefs and have the largest declines over time except among Asian students. To test this, the intercept and slope of each science identity trajectory were regressed onto dichotomous indicators representing three of the four groups defined by gender and college generation status. Because the variance of the slope was only statistically significant for White students, the slope was regressed onto the intersectional identities only for White students. Statistically significant findings are discussed below along with examples of the non-significant findings; all findings are in Table 4.

**Table 4:** Linear growth curves with intersectional predictors

	Black		Latinx		Asian		White	
	Intercept *B* (SE)	Slope *B* (SE)	Intercept *B* (SE)	Slope *B* (SE)	Intercept *B* (SE)	Slope *B* (SE)	Intercept *B* (SE)	Slope *B* (SE)
Predictors (Reference group = Male PCG students)								
Female PFG students	−0.20 (0.09)*	—	−0.20 (0.07)**	—	−0.20 (0.12)	—	−0.25 (0.03)***	0.01 (0.01)
Female PCG students	−0.15 (0.09)^+^	—	−0.10 (0.09)	—	−0.18 (0.09)^+^	—	−0.16 (0.02)***	−0.00 (0.01)
Male PFG students	−0.16 (0.10)	—	−0.09 (0.08)	—	−0.28 (0.11)*	—	−0.18 (0.03)***	0.02 (0.01)**
Science grade (8^th^)	0.23 (0.03)***	—	0.26 (0.03)***	—	0.29 (0.05)***	—	0.30 (0.01)***	−0.03 (0.00)***
Predictors (Reference group = Female PFG students)								
Female PCG students	0.05 (0.08)	—	0.10 (0.06)	—	0.01 (0.10)	—	0.10 (0.03)***	−0.01 (0.01)*
Male PFG students	0.04 (0.08)	—	0.11 (0.06)^+^	—	−0.08 (0.12)	—	0.07 (0.04)^+^	0.01 (0.01)^+^
Science grade (8^th^)	0.23 (0.03)***	—	0.26 (0.03)***	—	0.29 (0.05)***	—	0.30 (0.01)***	−0.03 (0.00)***
Predictors (Reference group = Male PFG students)								
Female PCG students	0.01 (0.10)	—	−0.01 (0.06)	—	0.10 (0.09)	—	0.03 (0.03)	−0.03 (0.01)***
Science grade (8^th^)	0.23 (0.03)***	—	0.26 (0.03)***	—	0.29 (0.05)***	—	0.30 (0.01)***	−0.03 (0.00)***

*Note*. Due to the nonsignificant findings of variance around the slopes for Black, Latinx, and Asian students, the slope of the science identity trajectories was regressed onto the intersectional identities only for White students. PFG = potential first-generation college students; PCG = potential continuing-generation college students

SOURCE: U.S. Department of Education, Institute of Education Sciences, National Center for Education Statistics, High School Longitudinal Study of 2009 (HSLS:09), Base Year, First Year Follow-Up, Second Year Follow-Up

^*+*^*p* < 0.10. *p < 0.05. **p < 0.01. *p < 0.001

Among Black students, two comparisons were statistically significant when comparing the groups defined by gender and college generational status. Black male potential continuing-generation students had higher 9^th^ grade science identities than Black female potential first- (*B* = −0.20, SE = 0.09, *p* = 0.02) and, at the trend level, continuing-generation students (*B* = −0.15, SE = 0.09, *p* = 0.07). All other comparisons among Black students were not statistically significant, suggesting students had similar 9^th^ grade science identities; for example, Black female potential continuing- and first-generation college students had similar 9^th^ grade science identities. In sum, the findings partially supported the hypothesis that male potential continuing-generation students had stronger science identities whereas female potential first-generation students had the weakest. Contrary to the hypothesis, female potential first-generation students were similar to male potential first-generation students and female potential continuing-generation students.

Among Latinx students, two comparisons were statistically significant. Latina female potential first-generation students had lower science identity beliefs in 9^th^ grade compared to both Latino male potential continuing- (*B* = −0.20, SE = 0.07, *p* = 0.004) and, at the trend level, first-generation students (*B* = 0.11, SE = 0.06, *p* = 0.08). These findings partially supported the hypothesis as differences emerged between female potential first-generation students and male potential continuing-generation Latinx students. All other comparisons were non-significant; for example, Latina and Latino continuing-generation college students had similar 9^th^ grade science identities as did Latina and Latino first-generation college students.

For Asian students, two differences emerged among the groups. Asian male potential continuing-generation students had higher 9^th^ grade science identities than their first-generation counterparts (*B* = −0.28, SE = 0.11, *p* = 0.01) and, at the trend level, Asian female potential continuing-generation students (*B* = −0.18, SE = 0.09, *p* = 0.06). All other comparisons were non-significant. In support of the hypothesis for Asian students, female potential first-generation students did not differ in their science identities compared to male potential continuing-generation students (*B* = −0.20, SE = 0.12, *p* = 0.11).

Several comparisons among the four groups were statistically significant for White students. White male potential continuing-generation students had higher 9^th^ grade science identities than their male potential first-generation counterparts (*B* = −0.18, SE = 0.03, *p* < 0.001) and female potential first- (*B* = −0.25, SE = 0.03, *p* < 0.001) and continuing-generation students (*B* = −0.16, SE = 0.02, *p* < 0.001). Moreover, female potential continuing-generation students had stronger science identities than female potential first-generation students in 9^th^ grade (*B* = 0.10, SE = 0.03, *p* < 0.001). A trend level gender difference emerged among potential first-generation students in terms of science identity beliefs in 9^th^ grade, such that male potential first-generation students had stronger science identities compared to female potential first-generation students (*B* = 0.07, SE = 0.04, *p* = 0.06). All other comparisons were non-significant.

When analyzing the rate of growth of science identity over time among White students, four significant comparisons emerged. Male potential first-generation students had larger increases over time compared to their male potential continuing-generation peers (*B* = 0.02, SE = 0.01, *p* = 0.001). Parallel to males, female potential first-generation students (*B* = −0.01, SE = 0.01, *p* = 0.03) had larger increases in their science identity beliefs over time compared to female potential continuing-generation students. Also, there was a trend for male potential first-generation students (*B* = 0.01, SE = 0.01, *p* = 0.07) to have larger increases in science identity beliefs over time compared to female potential first-generation students. Lastly, compared to male potential first-generation students, female potential continuing-generation students had slower growth in their science identity beliefs over time (*B* = −0.03, SE = 0.01, *p* < 0.001).

### Robustness Checks

The robustness check, which include participants who participated in the study at the last time point, revealed a similar pattern of findings as the main study findings (see Appendix C). For all groups, the linear model was a better fit compared to the no growth model (Table 11). Analyses addressing the second research question revealed the same findings for Black, Latinx and White students as the original analyses (Table 12). However, there were some differences that emerged among Asian students compared to the original findings. The robustness check revealed that female potential first-generation students had weaker science identities in 9^th^ grade compared to male potential continuing-generation students (*B* = −0.29, SE = 0.12, *p* = 0.02), which was not statistically significant in the original findings. All other comparisons remained the same as the original findings among Asian students.

## Discussion

Science identity is important to study because it relates to factors such as pursuing science majors (Hazari et al., 2013), developing critical thinking skills, and developing science literacy (Vieira et al., 2011). Most studies examining race and ethnicity within science focus on disparities between groups and ignore the rich variability within each group (Causadias et al., 2018). Complementary work illustrating the spaces of diversity and similarity within each racial/ethnic group can help highlight who is pursuing science and groups that have been historically invisible in prior research. Additionally, most prior literature on science has overlooked college generation status and that demographic differences, such as gender differences, may vary by racial/ethnic group (Hsieh et al., 2021). The present study explored the developmental trajectories of science identity beliefs among a diverse and nationally representative sample of Black, Latinx, Asian, and White students from 9^th^ grade to three years post-high school to better understand who has stronger science identities and who could potentially benefit from more support within each racial/ethnic group.

### The Development of Students’ Science Identity Beliefs

Contrary to this study’s hypothesis, students’ science identity beliefs increased over time. Students went from largely disagreeing to slightly agreeing when asked if they identified with being a science person. Research suggests that students’ motivational beliefs, including their identity, generally decline or remain stable from middle school through high school (Wigfield & Cambria, 2010; Wigfield et al., 2006). In contrast, studies on college students have noted that the developmental changes of science identity vary, with some students experiencing increases whereas other students’ science identities remain stable (Robinson et al., 2018). These mixed findings on science identity over time among college students, as well as the findings of the current study, theoretically align with situated expectancy-value theory, which also highlights how students’ science identity may be strengthened, remain stable, or weaken over time based on their experiences (Eccles & Wigfield, 2020).

The current findings may also vary from prior work on high school students due to historical differences. In recent years, several interventions and programs have been developed to improve youth’s STEM motivational beliefs and persistence, especially among underrepresented populations (National Science Foundation, 2019). For example, the designation of first-generation college student and related institutional support structures is a relatively recent development (Azmitia et al., 2018). Students today may see more diversity within science and therefore may see themselves and may feel perceived by others as scientists. In addition to increased diversity within science, students may also be more engaged in science if teachers are including more active learning strategies and emphasizing the utility of science to students’ lives (Schmidt et al., 2019). Thus, how science content is delivered by teachers as well as the increased opportunities to engage in science (e.g., science-based out-of-school activities; Chittum et al., 2017) may be related to the results found in this study. Finally, increasingly diverse science curricula, such as innovative classes on forensic science and sports medicine, affords a diverse array of science career and pathways options students could find valuable and can envision themselves pursuing.

### Science Identity Differences Within Racial/Ethnic Groups

An important part of this study was to understand differences among the four groups at the intersection of gender and college generational status within each racial/ethnic group. In partial support of the hypothesis, female potential first-generation students had weaker science identities at 9^th^ grade compared to male potential continuing-generation students for Black, Latinx, and White students, though not Asian students. These differences emerged even after considering students’ science performance in 8^th^ grade, which was significantly associated with their science identity. Past studies outline how individuals who are members of multiple intersecting marginalized groups, such as female potential first-generation students, can fair worse on academic outcomes and motivational beliefs compared to those who are in a more privileged positions, such as being a boy and a potential continuing-generation student in science (Leaper et al., 2012; Snodgrass Rangel et al., 2020). These findings emphasize the need to support female first-generation students and to provide resources aimed at overcoming both gender-related (Starr, 2018) and first-generation related challenges (Gibbons and Borders, 2010) highlighted in the literature.

Female potential first-generation students also had lower identities than their female potential continuing-generation student counterparts among Whites, however these two groups were similar among Blacks, Latinxs, and Asians. Regardless of college generation status, female students from racial/ethnic minority backgrounds may encounter challenges related to racial/ethnic stereotypes that White female students may not face. This is well-documented in previous studies that highlight the disadvantage girls and women have due to the additional barriers, challenges, and discrimination they face related to their racial/ethnic backgrounds (Harackiewicz et al., 2016; Wilson & Kittleson, 2013). Future studies should further investigate what processes might account for such differences in women’s science identity beliefs. For example, qualitative studies may uncover themes that emerge from the unique experiences of female students of color, including Asian female students who encounter positive stereotypes in STEM compared to Black and Latinx students.

Another contribution of this study was the finding that among White students, male and female potential first-generation students’ science identities experienced larger increases over time compared to their potential continuing-generation students counterparts. Potential first-generation students may have greater room for improvement due to disparities they may experience (Engle, 2007) and have positive experiences that relate to their science motivational beliefs (Eccles & Wigfield, 2020). Because first-generation students are known to experience challenges and lack certain resources, universities may provide them with resources, such as a first-generation resource center or mentoring programs. These additional resources and support may support larger increases in science identities as they may create more positive experiences for first-generation students. Additionally, these resources have developed and grown in the past decade and may in part explain why there were increases rather than decreases in science identity beliefs over time.

### Limitations and Future Directions

The current study gives rise to new research questions and topics that should be further explored in future studies. Although our study highlights that science identity beliefs differ based on the intersection of gender and generational status for some groups, it does not explore the processes supporting these differences and whether the processes accounting for these differences differ by racial/ethnic group. Future studies can build upon this by exploring the processes that contribute to group differences, as well as why some groups had differences whereas others did not. Such research would help identify the specific support that might benefit each group. Another limitation was that only the average linear changes in students’ science identity beliefs were identified. Although this research aim provided informative findings, the next step would be to use mixed method approaches to further understand the heterogenous patterns of change over time and if there are specific turning points that launch students onto a different trajectory. Finally, the current study was not able to incorporate indicators of privilege or disadvantage since these were not present in the dataset. Future studies that focus on marginalized groups should incorporate these indicators in order to have a more in-depth analysis of specific ways in which privilege and discrimination play a role in individuals’ lives within science.

## Conclusion

More studies are needed on the within-group variability in science motivation and its development across diverse groups. Contrary to the literature, the findings indicated that science identity beliefs increased over time for all racial/ethnic groups examined. Further, students’ science identity beliefs varied by gender and college generational status. In every racial/ethnic group except Asians, female potential first-generation students had lower identities at 9^th^ grade compared to male potential continuing-generation students, who were hypothesized to have the lowest and highest identity beliefs, respectively. Also in every group, male potential first-generation students had similar 9^th^ grade science identities compared to female potential continuing-generation students. Among Black and Asian students, male potential continuing-generation students had higher science identity beliefs at 9^th^ grade than most other groups. Even though White potential first-generation students had lower 9^th^ grade beliefs than their continuing-generation peers for both male and female students, the growth in their identity beliefs was larger over time. Exploring the experiences of students in science, particularly during adolescence and emerging adulthood can further refine resources and supports for students that are marginalized or underrepresented in science. Moreover, the findings reflect the need to study college generational processes prior to when students reach college when they are more established, as there were differences as early as 9^th^ grade. Study findings emphasize the need for scholars and educators to consider the differing identities and experiences students have when creating interventions aimed at increasing science identity beliefs and other motivational beliefs.

## Appendix A

Comparisons of Samples

Table 5

**Table 5:** Comparisons between the excluded and analytic samples

	Analytic Sample			Excluded Sample			*t* test or Chi-square test	Effect size
Study variables	*N*	*M* (SE)/%	Min/Max	*N*	*M* (SE)/%	Min/Max		
Female students	21,170	49%	0/1	3,990	48%	0/1	14.04***	−0.02^b^
Race/ethnicity	21,170	—	1/5	3,080	—	1/5	254.25***	0.10^b^
Science identity (9^th^ grade)	21,080	2.31 (0.01)	1/4	4,030	2.13 (0.04)	1/4	6.46***	0.14^a^
Science identity (11^th^ grade)	20,780	2.48 (0.01)	1/4	—	—	1/4	—	—
Science identity (3 years post-HS)	20,210	2.61 (0.01)	1/4	3,930	2.49 (0.05)	1/4	1.35	0.05^a^
Science grade (8^th^ grade)	20,660	4.10 (0.02)	1/5	4,000	3.65 (0.07)	1/5	19.03***	0.42^a^

*Note*. Frequencies displayed are weighted for both samples. ^a^Indicates Cohen’s d was used for measuring effect size among independent sample *t-*tests for continuous variables. Standard interpretation: small effect: 0.20, moderate effect: 0.50, large effect: 0.80. ^b^Indicates Cramer’s V was used for measuring effect size among Chi-square tests for categorical variables. Standard interpretation: small effect: 0.10, moderate effect: 0.30, large effect: 0.50

SOURCE: U.S. Department of Education, Institute of Education Sciences, National Center for Education Statistics, High School Longitudinal Study of 2009 (HSLS:09), Base Year, First Year Follow-Up, Second Year Follow-Up

**p* < 0.05. ***p* < 0.01. ****p* < 0.001

Table 6

**Table 6:** Comparisons between complete data and missing data samples within the analytic sample for this study

	Complete Data Sample			Missing Data Sample			*t* test or Chi-square test	Effect size
Study variables	*N*	*M* (SE)/%	Min, Max	*N*	*M* (SE)/%	Min/Max		
Female students	12, 710	50%	0,1	8,460	47%	0/1	86.49***	−0.06^b^
Race/ethnicity	12,710	—	1,5	8,460	—	1/5	43.02***	0.05^b^
Potential first-gen. college	12,710	44%	0,1	8,460	57%	0/1	198.80***	0.10^b^
Family income	12,710	4.30 (0.04)	1,13	8,460	3.82 (0.09)	1/13	11.00***	0.15^a^
Science identity (9^th^ grade)	12,710	2.32 (0.01)	1,4	8,370	2.23 (0.03)	1/4	8.69***	0.13^a^
Science identity (11^th^ grade)	12,710	2.49 (0.01)	1,4	8,070	2.45 (0.03)	1/4	8.36***	0.12^a^
Science identity (3 years post-HS)	12,710	2.62 (0.01)	1,4	7,500	2.52 (0.05)	1/4	5.03***	0.12^a^
Science grade (8^th^ grade)	12,710	4.12 (0.02)	1,5	7,950	3.96 (0.04)	1/5	14.79***	0.24^a^

*Note*. Frequencies displayed are weighted for both samples. ^a^Indicates Cohen’s d was used for measuring effect size among independent sample *t-*tests for continuous variables. Standard interpretation: small effect: 0.20, moderate effect: 0.50, large effect: 0.80. ^b^Indicates Cramer’s V was used for measuring effect size among Chi-square tests for dichotomous variables. Standard interpretation: small effect: 0.10, moderate effect: 0.30, large effect: 0.50

SOURCE: U.S. Department of Education, Institute of Education Sciences, National Center for Education Statistics, High School Longitudinal Study of 2009 (HSLS:09), Base Year, First Year Follow-Up, Second Year Follow-Up

**p* < 0.05. ***p* < 0.01. ****p* < 0.001

## Appendix B

Measurement Invariance Tests

Table 7

**Table 7:** Measurement invariance tests of science identity latent growth curves for Black students

*Time invariance*										
Invariance Model	Robust χ^2^ goodness of fit			DIFFTEST			CFI	ΔCFI	RMSEA	ΔRMSEA
	Value	df	*p*	Value	df	*p*				
Configural	15.73	2	0.0004	—	—	—	0.999	—	0.057	—
Weak	11.42	4	0.02	0.06	2	0.97	1.00	0.001	0.029	0.028
Strong	29.02	10	0.001	17.98	6	0.006	0.999	0.001	0.030	0.001
*Gender invariance*										
Configural	20.10	4	0.0005	—	—	—	0.999	—	0.061	—
Weak	22.33	7	0.002	6.29	3	0.10	0.999	<0.001	0.045	0.016
Strong	41.11	16	0.0005	21.13	9	0.01	0.999	<0.001	0.038	0.007
*College generational status invariance*										
Configural	12.54	2	0.002	—	—	—	1.00	—	0.070	—
Weak	17.85	5	0.003	6.27	3	0.10	0.999	0.001	0.049	0.021
Strong	23.45	14	0.05	9.02	9	0.44	1.00	0.001	0.025	0.024

SOURCE: U.S. Department of Education, Institute of Education Sciences, National Center for Education Statistics, High School Longitudinal Study of 2009 (HSLS:09), Base Year, First Year Follow-Up, Second Year Follow-Up

Table 8

**Table 8:** Measurement invariance tests of science identity latent growth curves for Latinx students

*Time invariance*										
Invariance Model	Robust χ^2^ goodness of fit			DIFFTEST			CFI	ΔCFI	RMSEA	ΔRMSEA
	Value	df	*p*	Value	df	*p*				
Configural	8.87	2	0.01	—	—	—	1.00	—	0.032	—
Weak	7.87	4	0.10	0.94	2	0.63	1.00	<0.001	0.017	0.015
Strong	74.84	10	<0.001	59.69	6	<0.001	0.999	0.001	0.044	0.027
Partial Strong^a^	27.59	6	0.0001	15.81	2	0.0004	1.00	<0.001	0.033	0.016
*Gender invariance*										
Configural	33.08	12	0.001	—	—	—	1.00	—	0.033	—
Weak	30.59	15	0.01	1.34	3	0.72	1.00	<0.001	0.025	0.008
Strong	86.54	24	<0.001	47.30	9	<0.001	0.999	0.001	0.040	0.015
Partial Strong^b^	69.84	23	<0.001	33.96	8	<0.001	0.999	0.001	0.035	0.010
*College generational status invariance*										
Configural	12.14	4	0.02	—	—	—	1.00	—	0.035	—
Weak	12.58	7	0.08	2.87	3	0.41	1.00	<0.001	0.022	0.013
Strong	22.38	16	0.13	11.20	9	0.26	1.00	<0.001	0.016	0.006

*Note*. ^a^One threshold for indicator 1 and one threshold for indicator 2 were freely estimated across time. ^b^One threshold for indicator 1 was freely estimated across groups

SOURCE: U.S. Department of Education, Institute of Education Sciences, National Center for Education Statistics, High School Longitudinal Study of 2009 (HSLS:09), Base Year, First Year Follow-Up, Second Year Follow-Up

Table 9

**Table 9:** Measurement invariance tests of science identity latent growth curves for Asian students

*Time invariance*										
Invariance Model	Robust χ^2^ goodness of fit			DIFFTEST			CFI	ΔCFI	RMSEA	ΔRMSEA
	Value	df	*p*	Value	df	*p*				
Configural	6.64	2	0.04	—	—	—	1.00	—	0.037	—
Weak	7.38	4	0.12	2.13	2	0.34	1.00	<0.001	0.022	0.015
Strong	37.68	10	<0.001	28.38	6	0.0001	0.999	0.001	0.040	0.018
Partial Strong^a^	16.13	8	0.04	8.68	4	0.07	1.00	<0.001	0.025	0.003
*Gender invariance*										
Configural	7.09	4	0.13	—	—	—	1.00	—	0.030	—
Weak	15.86	7	0.03	8.19	3	0.04	1.00	<0.001	0.039	0.009
Strong	40.38	16	0.0007	24.42	9	0.004	0.999	0.001	0.042	0.003
*College generational status invariance*										
Configural	44.50	12	<0.001	—	—	—	0.999	—	0.057	—
Weak	39.18	15	0.001	0.43	3	0.93	0.999	<0.001	0.044	0.013
Strong	66.60	24	<0.001	27.56	9	0.001	0.998	0.001	0.046	0.002

*Note*. ^a^One threshold for indicator 1 was freely estimated across time

SOURCE: U.S. Department of Education, Institute of Education Sciences, National Center for Education Statistics, High School Longitudinal Study of 2009 (HSLS:09), Base Year, First Year Follow-Up, Second Year Follow-Up

Table 10

**Table 10:** Measurement invariance tests of science identity latent growth curves for white students

*Time invariance*										
Invariance Model	Robust χ^2^ goodness of fit			DIFFTEST			CFI	ΔCFI	RMSEA	ΔRMSEA
	Value	df	*p*	Value	df	*p*				
Configural	21.18	2	<0.001	—	—	—	1.00	—	0.029	—
Weak	22.93	4	0.0001	6.38	2	0.04	10.00	<0.001	0.020	0.009
Strong	202.58	10	<0.001	159.10	6	<0.001	0.999	0.001	0.041	0.021
Partial Strong^a^	85.41	8	<0.001	56.20	4	<0.001	1.00	<0.001	0.029	0.009
*Gender invariance*										
Configural	36.50	4	<0.001	—	—	—	1.00	—	0.037	—
Weak	30.80	7	0.0001	4.44	3	0.22	1.00	<0.001	0.024	0.013
Strong	107.47	16	<0.001	72.51	9	<0.001	1.00	<0.001	0.031	0.007
*College generational status invariance*										
Configural	23.95	4	0.0001	—	—	—	1.00	—	0.029	—
Weak	31.34	7	0.0001	11.19	3	0.01	1.00	<0.001	0.024	0.005
Strong	62.66	16	<0.001	34.93	9	0.0001	1.00	<0.001	0.022	0.002

*Note*. ^a^One threshold for indicator 2 was freely estimated across time

SOURCE: U.S. Department of Education, Institute of Education Sciences, National Center for Education Statistics, High School Longitudinal Study of 2009 (HSLS:09), Base Year, First Year Follow-Up, Second Year Follow-Up

## Appendix C

Robustness Checks

Table 11

**Table 11:** Model fit comparisons for the latent growth curve analyses of science identity by racial/ethnic group from 9^th^ grade to 3 years post-HS (robustness check)

*Black students’ science identity*											
Model	χ^2^	df	*p*	Δ χ^2^	Δdf	*p*	RMSEA	90% CI	CFI	TLI	SRMR
No growth	125.40	6	<0.001	—	—	—	0.116	0.099 0.134	0.430	0.715	0.136
Linear*	13.90	3	<0.001	115.50	3	**<0.001**	0.049	0.025 0.077	0.948	0.948	0.034
*Latinx students’ science identity*											
No growth	164.97	6	<0.001	—	—	—	0.108	0.094 0.123	0.590	0.795	0.123
Linear*	24.89	3	<0.001	140.08	3	**<0.001**	0.057	0.038 0.078	0.944	0.944	0.054
*Asian students’ science identity*											
No growth	41.65	6	<0.001	—	—	—	0.068	0.050 0.088	0.761	0.881	0.102
Linear*	14.22	3	<0.01	27.43	3	**<0.001**	0.054	0.028 0.084	0.925	0.925	0.082
*White students’ science identity*											
No growth	684.98	6	<0.001	—	—	—	0.116	0.109 0.123	0.651	0.825	0.102
Linear*	142.91	3	<0.001	542.07	3	**<0.001**	0.074	0.064 0.085	0.928	0.928	0.053

*Note*. The robustness check sample was composed of excluded participants who did not participate in the last time point (i.e., when students were three years post-high school). The total sample size for the robustness check was 14,890. *Indicates the model with the best fit indices

SOURCE: U.S. Department of Education, Institute of Education Sciences, National Center for Education Statistics, High School Longitudinal Study of 2009 (HSLS:09), Base Year, First Year Follow-Up, Second Year Follow-Up

Table 12

**Table 12:** Linear growth curves with intersectional identity predictors (robustness check)

	Black		Latinx		Asian		White	
	Intercept *B* (SE)	Slope *B* (SE)	Intercept *B* (SE)	Slope *B* (SE)	Intercept *B* (SE)	Slope *B* (SE)	Intercept *B* (SE)	Slope *B* (SE)
Predictors (Reference group = Male PCG students)								
Female PFG students	−0.20 (0.09)*	—	−0.19 (0.07)*	—	−0.29 (0.12)*	—	−0.25 (0.03)***	0.01 (0.01)
Female PCG students	−0.16 (0.09)^+^	—	−0.11 (0.09)	—	−0.18 (0.10)^+^	—	−0.15 (0.03)***	−0.00 (0.01)
Male PFG students	−0.15 (0.11)	—	−0.09 (0.08)	—	−0.22 (0.11)*	—	−0.19 (0.03)***	0.03 (0.01)***
Science grade (8^th^ grade)	0.23 (0.03)***		0.26 (0.03)***		0.33 (0.04)***		0.30 (0.01)***	−0.02 (0.00)***
Predictors (Reference group = Female PCG students)								
Female PCG students	0.04 (0.08)	—	0.09 (0.07)	—	0.12 (0.10)	—	0.10 (0.03)***	−0.01 (0.01)*
Male PFG students	0.04 (0.10)	—	0.11 (0.06)^+^	—	0.08 (0.12)	—	0.06 (0.04)	0.02 (0.01)*
Science grade (8^th^ grade)	0.23 (0.03)***		0.26 (0.03)***		0.33 (0.04)***		0.30 (0.01)***	−0.02 (0.00)***
Predictors (Reference group = Male PFG)								
Female PCG students	−0.00 (0.11)	—	−0.02 (0.07)	—	0.04 (0.10)	—	0.05 (0.03)	−0.03 (0.01)***
Science grade (8^th^)	0.23 (0.03)***	—	0.26 (0.03)***	—	0.33 (0.04)***	—	0.30 (0.01)***	−0.02 (0.00)***

*Note*. The robustness check sample was composed of excluded participants who did not participate in the last time point (i.e., when students were three years post-high school). The total sample size for the robustness check was 14,890. Due to the nonsignificant findings of variance around the slopes for Black, Latinx, and Asian students we regressed the slope of the science identity trajectories onto the intersectional identities only for White students. PFG = potential first-generation college students; PCG = potential continuing-generation college students

SOURCE: U.S. Department of Education, Institute of Education Sciences, National Center for Education Statistics, High School Longitudinal Study of 2009 (HSLS:09), Base Year, First Year Follow-Up, Second Year Follow-Up

**p* < 0.05. ***p* < 0.01. **p* < 0.001
